# Spatial variation, pooled prevalence, and factors associated with perinatal mortality in Sub-Saharan Africa, evidence from demographic and health surveys 2015–2023: a geospatial regression approach

**DOI:** 10.1016/j.eclinm.2025.103137

**Published:** 2025-03-06

**Authors:** Belayneh Jejaw Abate, Alemakef Wagnew Melesse, Helen Brhan, Muluken Chanie Agimas

**Affiliations:** Department of Epidemiology and Biostatistics, Institute of Public Health, College of Medicine and Health Sciences, University of Gondar, Gondar, Ethiopia

**Keywords:** Perinatal mortality, Spatial analysis, Multi-scale geographic weighted regression, Sub-Saharan Africa

## Abstract

**Background:**

Sub-Saharan Africa (SSA) bears the greatest burden of perinatal mortality in the world, and the magnitude of the problem varied based on geographical location. A detailed understanding of spatial variation is important to improve the targeting of interventions, to identify the most affected community, and for designing evidence-based health policies. Hence, this study aimed to assess pooled prevalence, spatial variation, and factors contributing to perinatal mortality in SSA.

**Methods:**

A cross-sectional study using Demographic and Health Survey datasets (2015–2023) of 25 SSA countries with a total of 201,566 weighted samples was used for this study. The global spatial autocorrelation was explored using global Moran’s-I, and the spatial variation of perinatal mortality was examined using hot spot analysis (Local Getis-Ord Gi∗ statistic). Spatial regression analyses (ordinary least squares, spatial error model, spatial lag model, geographically weighted regression, and multiscale geographically weighted regression) were conducted. Models were assessed using corrected Akaike information criteria and adjusted R^2^. A p-value threshold of 0.05 was set to identify statistically significant spatial predictors, and the corresponding local coefficients were illustrated on a map.

**Findings:**

The pooled prevalence of perinatal mortality in SSA was 46.63 per 1000 total births (95% CI: 42.48, 51.17), and its spatial distribution was found to be clustered (Global Moran’s I = 0.18, p < 0.01). Significant hotspot areas were located in Nigeria, Madagascar, Rwanda, Malawi, Burundi, Gambia, Uganda, Côte d’Ivoire, Angola, Ethiopia, Burkina Faso, and Senegal, while significant cold spots were located in Kenya, Gabon, South Africa, Ghana, Mali, and Mauritania. The multi-scale geographic weighted regression model explained 85% of the spatial variation of perinatal mortality in SSA. No antenatal care visit, birth interval less than 15 months, women undergoing cesarean section delivery, unemployed women, and households without children were significant spatial predictors of perinatal mortality in SSA.

**Interpretation:**

Perinatal mortality in SSA was high and varied across regions. We identified five predictors for perinatal mortality that might be a priority for policymakers. Enhancing antenatal care and family planning services and empowering women through employment opportunities is crucial to decreasing perinatal mortality in the region.

**Funding:**

None.

**Research in context**

**Evidence before this study**

SSA bears the greatest burden of perinatal mortality in the world. This study aimed to determine pooled prevalence, geospatial variation, and factors associated with perinatal mortality in SSA. We searched PubMed using MeSH terms, phrases, and Boolean operators for published papers without any language restriction, using the terms “perinatal mortality,” OR “perinatal death,” OR “perinatal mortal,” AND “geospatial variation,” OR “geographical variation” AND “pooled prevalence” OR “overall magnitude” OR “combined prevalence” AND “associated factors” AND “SSA” NOT “comment” NOT “case report.” We found that one systematic review and numerous extensive studies have been released throughout SSA on the determinants of perinatal mortality. Nevertheless, these studies mainly relied on relative measures of association, such as odds ratios (ORs) or relative risks (RRs), and also, they don’t consider factors associated with perinatal mortality at varying spatial scales, which might make it difficult to identify the most affected community, to design local interventions, and to provide evidence for policy and decision-makers based on their local variation. To address these gaps, subnational estimates of perinatal mortality will be required, as such data has not yet been accessible for many countries.

**Added value of this study**

To the best of our understanding, this is the latest nationally representative DHS data set to identify pooled prevalence, geospatial variation, and factors associated with perinatal mortality in 25 SSA countries. Our finding revealed that the pooled prevalence of perinatal mortality in SSA was 46.63 per 1000 total births. We identified significant hotspot areas of perinatal mortality, which resided in Nigeria, Madagascar, Rwanda, Malawi, Burundi, Gambia, Uganda, Côte d’Ivoire, Angola, Ethiopia, and Burkina Faso. To overcome the limitation of previous studies, we employed a geospatial method, particularly MGWR, to identify factors associated with perinatal mortality with a varying spatial scale. No ANC visit, birth interval less than 15 months, women undergoing cesarean section delivery, unemployed women, and households without children were significant spatial predictors of perinatal mortality in SSA. Geographical differences in predictors were also observed across countries in Sub-Saharan Africa.

**Implications of all the available evidence**

The elevated rate of perinatal mortality implies a critical need for improved health care intervention in SSA. Our findings bring new evidence and suggest that national health policies should prioritize hotspot regions with higher perinatal mortality rates, allowing for strategic allocation of public health resources to those areas in need.

## Introduction

Enhancing child survival is a crucial priority in global health initiatives. Perinatal mortality, which is defined as stillbirths and deaths of newborns within the first week of life, is the most crucial phase for securing a child’s survival.^1^

Every year, 8 million perinatal deaths occur worldwide,^2^^,^^3^ which is approximately five times higher in low-income countries compared to high-income countries.^4^ Taking this into account, significant advancements were made through initiatives such as the Every Newborn Action Plan, Every Woman Every Child Monitoring Framework, Every Child Alive Campaign, and the Quality of Care Network. Those initiatives focus on the reduction of perinatal mortality in low- and middle-income countries and the successful achievement of Sustainable Development Goals.^5^ Despite global efforts to reduce perinatal mortality, it remains a significant challenge, especially in SSA.^6^

SSA bears the greatest burden of perinatal mortality in the world, accounting for 40% of total under-five deaths.^7^ A child born in SSA has a 1 in 36 likelihood of experiencing perinatal death, compared to 1 in 333 in high-income countries.^6^ Addressing these disparities remains a critical focus for health organizations worldwide.^4^

Previous studies from the SSA countries identified several predictors linked to higher perinatal mortality, including birth interval, preterm delivery, anemia, congenital anomalies, previous neonatal death, low birth weight, birth complications, infections, maternal conditions,^8^, ^9^, ^10^, ^11^ and also various determinants such as maternal education, place of residence, age, pregnancy history, and place of delivery.^7^ The impact of each cause varies geographically,^12^ while previous studies mainly employed relative measures of association, like odds ratios (ORs) and relative risks (RRs)^13^; however, relying on those measures results in ignoring geographic variability, difficulty in identifying spatial patterns, and an inability to effectively capture interaction between variables influenced by geographical factors, which ultimately is not an effective strategy for public health planning and resource allocation. As spatial analyses are important to identify the most affected community,^14^ design local interventions,^15^ allocate scarce resources to the most affected areas,^4^ and provide evidence for policy and decision-makers to ensure equity in the community.^7^^,^^16^ In addition, previous studies had performed spatial analysis for a single country, did not incorporate the pooled prevalence of SSA,^7^^,^^17^^,^^18^ and its associated factors at varying spatial scales^1^^,^^4^^,^^19^^,^^20^ between 2015 and 2023. Making it difficult to identify the recent magnitude, geographical variation among countries, and its predictors within a specified spatial scale, geospatial methods, particularly multi-scale geographic weighted regression (MGWR), are able to analyze data from a variety of spatial scales, hence providing flexibility in understanding the ways in which factors of influence change within the geographic context in which traditional statistical methods may fail to disclose. In light of this, our study aims to investigate the spatial distribution, pooled prevalence, and factors associated with perinatal mortality in SSA using geospatial analysis. This will lead to more accurate risk assessment, resource allocation, and intervention strategies based on local variation in the relationships between perinatal mortality and its determinants.

## Methods

### Study design and data sources

A community-based cross-sectional study was conducted using nationally representative Demographic and Health Survey data between 2015 and 2023 in 25 SSA countries.

Countries were chosen based on the accessibility of recent up-to-date datasets and categorized as Eastern SSA (Burundi, Ethiopia, Kenya, Malawi, Mozambique, Rwanda, Tanzania, Uganda, Zambia, Zimbabwe), Central SSA (Angola, Gabon), Western SSA (Burkina Faso, Ivory Coast, Gambia, Ghana, Guinea, Liberia, Mali, Mauritania, Nigeria, Senegal, Sierra Leone), and Southern SSA (Madagascar, South Africa).

The DHS surveys are carried out by the health ministry or governmental agencies in each country, supported by ICF International. These surveys include comprehensive and nationally representative studies that offer insights into various health and demographic indicators in low- and middle-income nations.^5^

### Ethics statement

After the consent paper was submitted to the DHS Program, a letter of permission was waived from the International Review Board of Demographic and Health Surveys (DHS) program data archivists to download datasets for those selected countries. This study followed the Strengthening the Reporting of Observational Studies in Epidemiology (STROBE) guidelines for cross-sectional studies (STROBE Checklist).^21^

### Sampling procedures and sample size

Participants for this study were chosen by using a two-stage stratified cluster sampling method. In the first stage, a total of 15,785 enumeration areas (EA) were selected with probability proportional to the size of EA. In the second stage, a fixed number of households were selected by using the equal probability systematic sampling method in the selected cluster. A total of 392,887 women aged 15 to 49 who were either permanent residents of the selected households or visitors who stayed overnight at those households before the data collection day were eligible for the interview. Finally, the study selected a weighted sample of 201,566 women who had delivered at seven or more months’ gestational age, including both stillbirth and live birth, and seven days postpartum (0–6 days) in the 5 years preceding the survey from the DHS Individual Records (IR) dataset of all 25 countries. Appendix 1 reveals the sampling procedure and sample size determination for each selected country survey.

### Outcome variables

The outcome variable “perinatal mortality” was determined by using procedures outlined in the DHS Contraceptive Calendar tutor^22^; according to the tutor, stillbirth was defined as the number of fetal deaths in pregnancy of seven or more months.^7^ Early neonatal death: defined as a death in the first seven days (days 0–6) of a child born alive.^7^ Perinatal mortality is defined as stillbirths plus early neonatal deaths in the five years preceding the survey divided by all births (including stillbirths) that had a pregnancy duration of seven or more months. Then dummy variables were generated for the outcome variable “perinatal mortality,” coded as “1” for “yes” and “0” for “no.” The details of the calculation method for the perinatal mortality rate were presented in Appendix 2.

### Explanatory variables

Potential explanatory variables were selected by previously published articles and categorized as socio-demographic characteristics (women’s age, sex of household head, women’s education level, father’s education level, and place of residence, number of children in the house, mother’s occupation, and wealth index), maternal related factors (Tetanus vaccine (TT), ANC visit, women access to decision making, age at first birth, and history of pregnancy ever terminated), obstetrics-related factor (place of delivery, delivery assistance, birth interval, and mode of delivery), environmental related factors (drinking water source, toilet facility, media exposure, type of cooking fuel, and distance from health facility). A comprehensive list of all predictors, including their measurements, is available in Appendix 2.

### Statistical analysis

Once selected variables are retained based on a literature review, the data were modified, edited, verified, arranged, and recoded in STATA 17 software. In this process, missing data were observed for different variables, like mother education (3.4%), father education (4.1%), place of delivery (2.96%), women undergoing caesarean section delivery (3.11%), ANC visit (4.5%), TT vaccine (3.3%), birth interval (6.4%), and distance from health facility (2.7%). The pattern of missing was arbitrary, considering that the pattern of missing was missing at random and managed according to the DHS guideline (Appendix 3).

To make the survey representative and to get reliable statistical estimates, the data were weighted using the STATA command “[iw = wgt].” The pooled prevalence of perinatal mortality was determined by the “Metaprop” statistical command; the presence of heterogeneity among the studies was examined using the I^2^ test and Cochrane Q statistics. Since the test statistics revealed a considerable heterogeneity among studies, Der Simonian and Laird’s random effects model was used to estimate the pooled effect. To account for heterogeneity, subgroup analysis was carried out based on regions of the SSA countries.

### Spatial analysis

In the first stage, the aggregated percentage data of perinatal mortality was linked to geographic coordinates using the unique identification code for each enumeration area (EA). Then maps were constructed by the Africa Albers Equal Area Projection based on the World Geodetic System 84 (WGS84) coordinate reference system; these were selected because the projected area of SSA was approximately equal to the true area of SSA (Appendix 4).

The spatial pattern of perinatal mortality was identified by several tests, including spatial autocorrelation to determine whether the spatial distribution of perinatal mortality was randomly distributed, clustered, or dispersed throughout SSA; hot spot analysis to examine “hotspots “and “cold spots” areas of perinatal mortality; however, since it does not detect outliers, cluster-outlier analysis was performed. Furthermore, spatial scan statistics and ordinary kriging interpolation were examined to detect significant clusters and to predict spatial variation of perinatal mortality in unsampled areas of SSA countries by using sampled areas, respectively. Kriging interpolation relies on several key assumptions, including the assumption of stationarity, which posits that the statistical properties of the underlying process do not change over space, isotropy: This assumes that spatial correlation is consistent in all directions. Additionally, it assumes spatial correlation between sampled points can be adequately described by a covariance function, allowing for predictions based on nearby observations. However, these tests do not reveal the geographical impact of each independent variable on the outcome, so we conduct spatial regression. We utilized five distinct spatial models, including three global models and two local models. Prior to constructing these regression models, we employed various mechanisms to achieve normality, such as log transformation, square root transformation, and Box-Cox transformation. Finally, Box-Cox transformations were selected, as they best fulfilled the underlying assumptions (Appendix 5).

Initially, Ordinary Least Squares (OLS) was applied to select variables that are suitable for analyzing the spatial variation in perinatal mortality. To identify a model that meets the assumptions of the OLS method, exploratory regression analysis was performed to identify variables that fulfill all of the assumptions required for the OLS method to be valid (Appendix 6). The OLS regression assumes stationarity, spatial independence of residuals, normality of errors, absence of multicollinearity, significance of predictors, and model performance. OLS also acted as a diagnostic tool to identify which predictors should be included in additional comparative models.

The global models assume uniform relationships across all areas, meaning that the effects of predictors on perinatal mortality are considered constant throughout the study region. For instance, OLS regression helps identify significant predictors while assuming spatial independence of residuals. However, this assumption may not hold in the presence of spatial autocorrelation, which is why spatial lag model (SLM) and spatial error model (SEM) were utilized. These models account for spatial dependencies among observations, allowing us to examine how perinatal mortality rates are influenced by neighboring regions.

Several tests were performed to evaluate the spatial dependency of the model; this study uses a specific spatial diagnostic test, which is the Lagrange multiplier (LM) test, that is important to determine the most appropriate model for the data. If the LM-lag and robust LM-lag tests yield significant values, it indicates that a spatial lag model is appropriate. Conversely, significant values from the LM-error and robust LM-error tests suggest the validity of a spatial error model (Appendix 7). The study identified the significant Lagrange multiplier tests (p < 0.001) for both lag and error effects, and then we applied SLM and SEM to see the spatial dependency between the perinatal mortality in neighbors and the spatial autocorrelation in the residuals, respectively. In addition, SEM takes into account unobserved spatial factors that influence the error terms in regression analysis, recognizing the impact of omitted variables on spatial dependence.

The Koenker Breusch–Pagan (BP) test was significant, indicating the presence of non-stationarity or heterogeneity in the relationship between perinatal mortality and predictors. As a result, Geographically Weighted Regression (GWR) was used to detect spatial neglect as it considers spatial non-stationarity. The most prominent limb of GWR is that it fits a localization-based regression model independently for each spatial unit and allows the regression coefficients to vary within the area of study. However, the GWR suffers from the assumption of coexistence of all the predictors on a similar spatial scale, which may not be accurate, as the optimal scale could differ for each predictor variable. This is important because, by allowing each predictor variable to operate at its own optimal spatial scale, the MGWR method solves a major limitation of the standard GWR approach. The bandwidth for each of the predictors is determined individually using the golden section search rather than assuming a uniform spatial scale for all the covariates. This approach allows the model to capture various scales of relationship non-stationarity for every target-to-predictor variable linkage,^23^ while MGWR also adopts an iterative back-fitting procedure when model calibration is considered, which extends the GWR framework to further account for different processes that may operate at different spatial scales within the study area.

MGWR can be formulated as

$$\text{yi}=\sum_{j=1}^{\;m}\beta \text{bwjXij}\;+\;\varepsilon i.\;i=1\;2\;3\ldots n$$

Where βbwj is the bandwidth used for calibration of the jth relationship, Xij is the value of the jth explanatory parameter, and εi is a random error term.

Finally, the same variables that had passed in the global model were used in the local model.

Models with the highest adjusted R^2^ and lowest corrected Akaike Information Criterion (AICc) were indicative of better model performance and also statistically significant (p < 0.01) spatial predictors, and their corresponding coefficients were displaced on the map. In our study, the unit of analysis for each model was at the level of enumeration areas (EAs) aggregated across 25 SSA countries (Appendix 8A). The global spatial models were executed in GeoDa 1.14, while the local spatial models were developed using ArcGIS Pro version 3.1. The working flow and description of the spatial regression model were depicted in Appendix 8B.

### Role of the funding source

There was no funding source for this study.

## Results

### Pooled prevalence of perinatal mortality in SSA

**Fig. 1:**
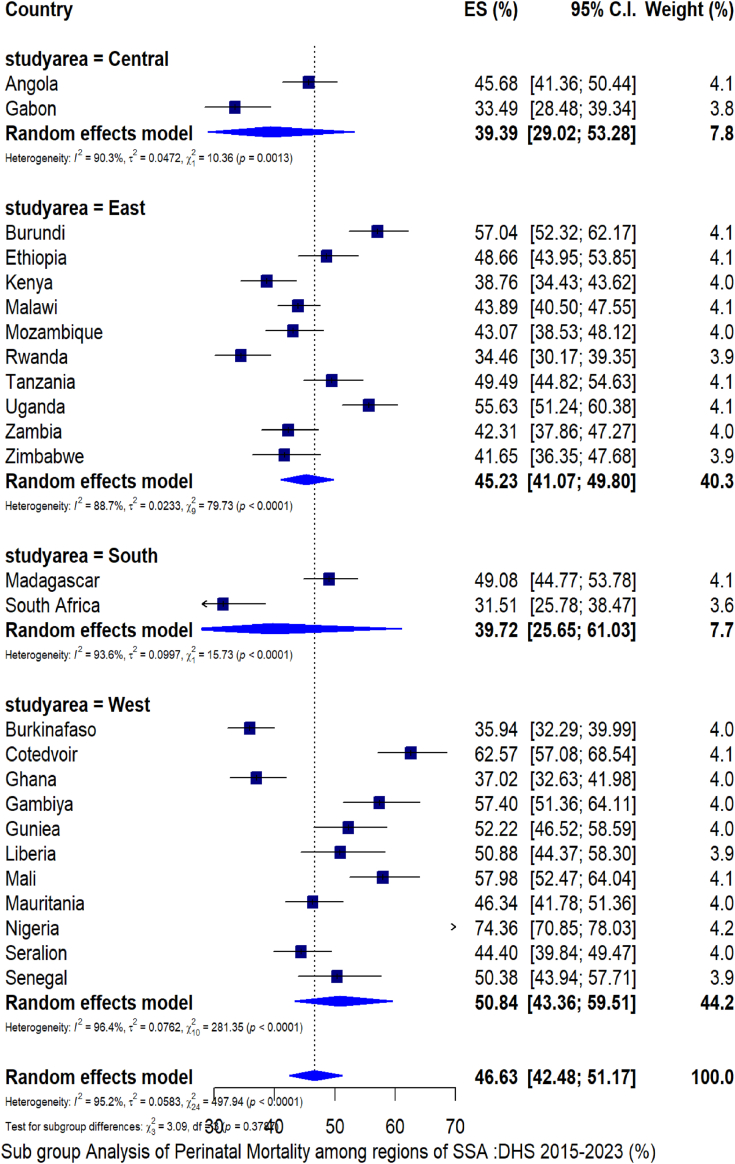
Pooled prevalence of perinatal mortality in Sub-Saharan Africa; DHS 2015–2023. Notes: Results are obtained from Der Simonian and Laird’s random effects model. Es denotes effect size, SSA denotes Sub-Saharan Africa, DHS denotes demographic and health survey data, and C.I. denotes confidence interval.

A total of 201,566 study participants from 25 SSA countries were included in the analysis. The pooled prevalence of perinatal mortality in Sub-Saharan Africa was 46.63 per 1000 total births (95% CI: 42.48, 51.17) with $I^{2}$ of 95%, p-value < 0.01. The rate was varied across sub-regions of SSA. Central SSA reported a rate of 39.39 per 1000 total births, with Angola leading at 45.68 per 1000 total births. Southern SSA followed closely with a pooled estimate of 39.72, where Madagascar recorded the highest rate at 49 per 1000 total births. Eastern SSA shows a higher rate of 45.2 per 1000 total births, with Burundi standing out at 57.04. Finally, Western SSA boasts the highest overall rate at 50.84 per 1000 total births, highlighted by Nigeria’s remarkable 74 per 1000 total births (Fig. 1).

### Global spatial autocorrelation (Moran’s I) analysis

The study found significant spatial clustering (Moran’s I 0.18, z-score 40.4, p-value < 0.01), revealing a 99% confidence level in the clustering pattern of perinatal mortality across the regions of SSA (Appendix 9). Further analysis confirmed this clustering with a z-score of 38.8 and a p-value less than 0.001, showing that high values are more concentrated than expected by chance (Appendix 9).

### Hot/cold spot area of perinatal mortality in SSA

**Fig. 2:**
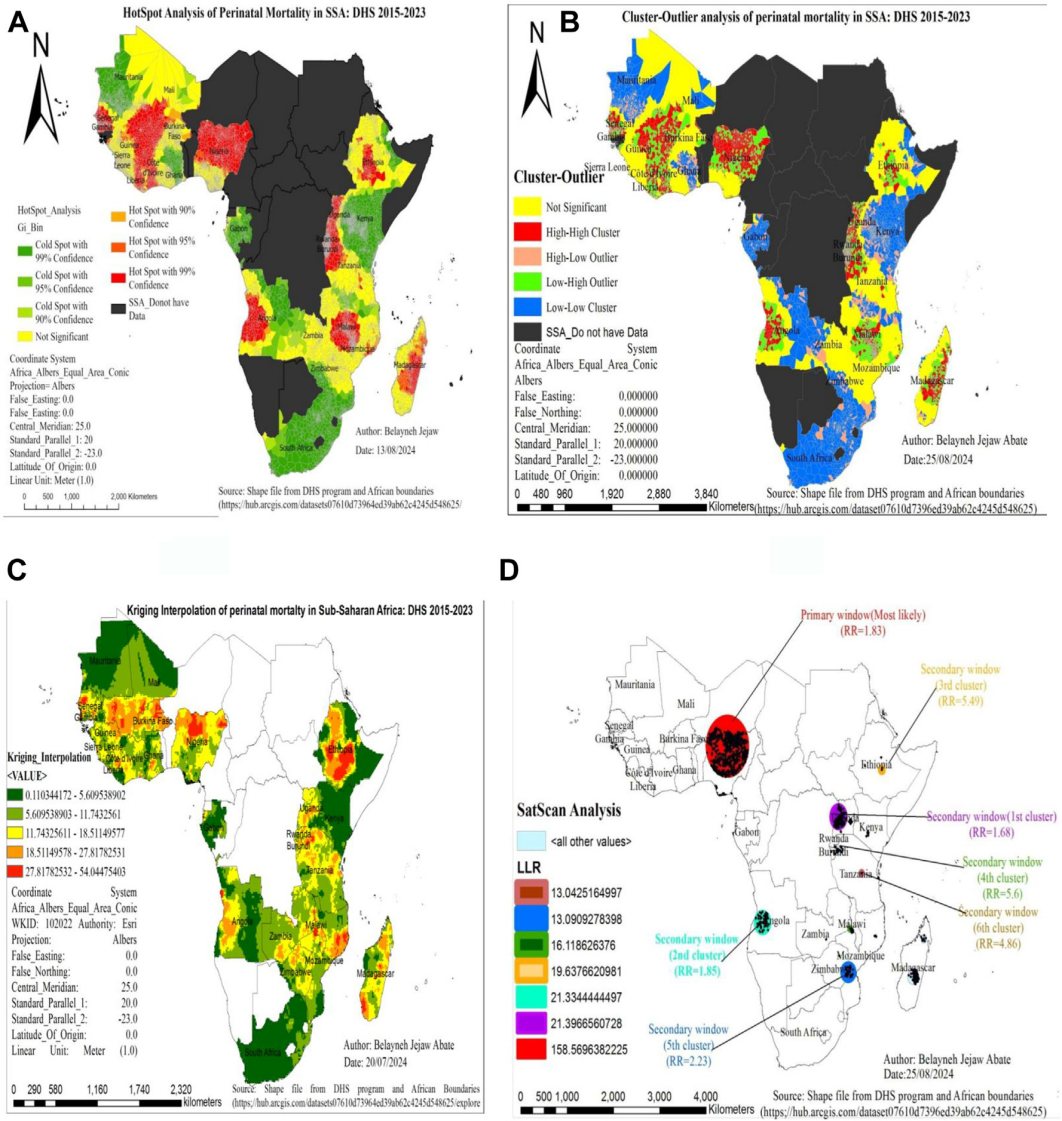
Hotspot (A), cluster-outlier (B), spatial interpolation (C), and SatScan (D) analysis of perinatal mortality in SSA: DHS 2015–2023. Notes: Red color shades in Fig. A, B, and C indicate countries with high perinatal mortality, and green color shades indicate the countries with low perinatal mortality. Strength of presence is indicated by depth of color. SSA denotes Sub-Saharan Africa, DHS denotes demographic and health survey data, C.I. denotes confidence interval, and LLR denotes log likelihood ratio.

Statistically significant hot spot areas of perinatal mortality were clustered in Nigeria, Madagascar, Rwanda, Malawi, Burundi, Gambia, Uganda, Côte d’Ivoire, Angola, Ethiopia, Burkina Faso, and Senegal. While Kenya, Gabon, South Africa, Ghana, Mali, and Mauritania were detected as cold spot areas (Fig. 2A).

### Cluster-outlier analysis of perinatal mortality in SSA

Significant high-high clusters were detected in Nigeria, Burundi, Côte d’Ivoire, Madagascar, Uganda, Senegal, Ethiopia, Rwanda, and Burkina Faso that are considered hotspots. In contrast, low-low significant clusters were reported in South Africa, Kenya, Gabon, Ghana, and Mauritania. The study also identified low-high outliers in Mozambique, Angola, and Malawi, as well as high-low outliers in Tanzania and Zambia (Fig. 2B).

### Spatial prediction of perinatal mortality in SSA

The highly predicted areas of perinatal mortality in SSA were reported in Uganda, Kenya, Tanzania, Madagascar, Nigeria, Burkina Faso, Côte d’Ivoire, Sierra Leone, Guinea, Senegal, Angola, Burundi, and Ethiopia, whereas Mauritania, South Africa, Mali, Kenya, and Gabon were predicted to have a low prevalence (Fig. 2C).

### SatScan analysis of perinatal mortality in SSA

A total of 924 significant clusters were detected using Kulldorff’s spatial scan statistic. Out of those, 678 were most likely clusters, and 246 were secondary clusters. The most likely primary cluster was located in Nigeria (11.19038°N, 7.8251136°E; 475.39 km radius), having a 1.83 times higher perinatal mortality rate compared to outside the spatial window (RR = 1.83, p-value < 0.001) (Fig. 2D). Secondary clusters were found in Uganda (−1.415116°N, 30.87648°E; 179.7 km radius, RR = 1.68, p < 0.01), Angola (−13.02157°N, 14.98804°E; 69.4 km radius, RR = 1.85, p < 0.01), Ethiopia (7.887302°N, 39.86272°E; 29.71 km radius, RR = 5.49, p < 0.01), Burundi (13.91513°N, 33.35088°E; 0 km radius, RR = 5.6, p < 0.01), Zimbabwe (20.02374°N, 33.01289°E; 142.9 km radius, RR = 2.23, p = 0.02), and Tanzania (−6.221925°N, 35.75617°E; 5.74 km radius, RR = 4.86, p = 0.02), all having significantly higher perinatal mortality rates within the clusters compared to outside the spatial window (Appendix 10).

### Spatial predictors of perinatal mortality in SSA

Perinatal mortality was spatially clustered (Moran’s I 0.18, z-score 40.4, p-value < 0.01) across regions of SSA, indicating a failure of the OLS assumption of spatial independence, leading us to employ a spatial dependence model. The significant Lagrange multiplier tests (p < 0.001) for both lag and error effects prompted two additional global spatial models (SLM and SEM) to better account for the underlying spatial dependence; all predictors were checked on the assumption of spatial dependence (Appendix 11). However, the significant Koenker-Basset statistics enabled us to perform local regression (GWR and MGWR). Among all models, MGWR can explain up to 85.6% of the variables that are causing perinatal mortality in SSA. This was supported by the dropping figure of AICc from 33887.8 for OLS to 16460.4 for MGWR. This indicates that MGWR is the most suitable model (Appendix 12).

**Fig. 3:**
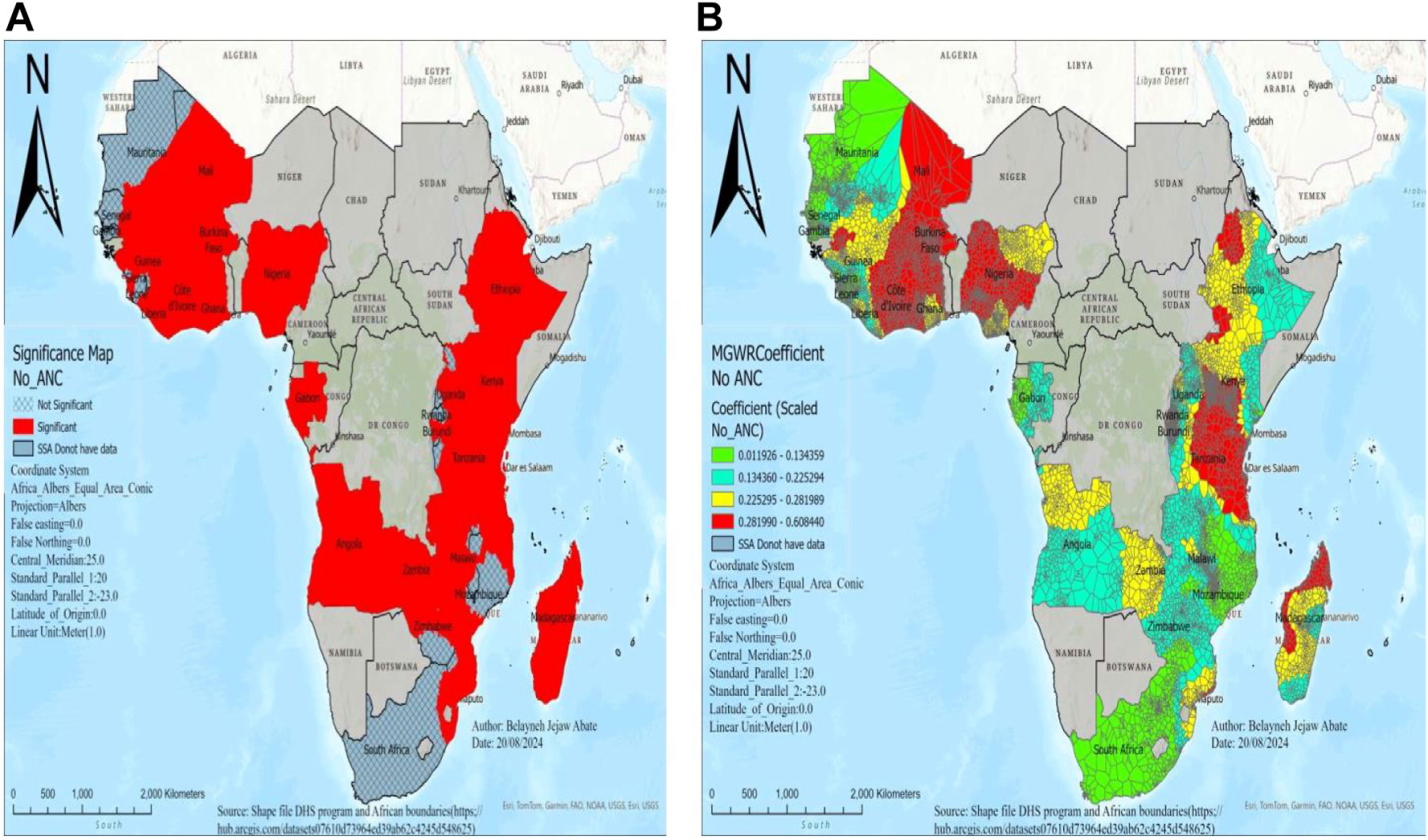
MGWR result of significant areas (A) and coefficients (B) of no ANC visit for estimating perinatal mortality in SSA DHS 2015–2023. Notes: Coefficient estimates and significance maps are obtained from MGWR regression. Covariates included are no antenatal care visit. Red color shades in (B) indicate countries with high perinatal mortality associated with no antenatal care visit, and green color shades indicate the countries with low perinatal mortality. Strength of presence is indicated by depth of color. ANC represents antenatal care, DHS denotes demographic and health survey data, and MGWR represents multi-scale geographic weighted regression.

No ANC visit had a positive effect on perinatal mortality in our final model (MGWR), which ranges from 0.01 to 0.6 and was statistically significant in areas of Ethiopia, Uganda, Kenya, Tanzania, Madagascar, Zambia, Angola, Gabon, Nigeria, Mali, Burkina Faso, Guinea, Ghana, Côte d’Ivoire, and Liberia. The highest statistically significant positive effect with a 10% increase in women receiving no ANC visit results in a 2.1%–6% rise in perinatal mortality in areas of Mali, Burkina Faso, Nigeria, Côte d’Ivoire, Ghana, and some parts of Ethiopia and Madagascar (Fig. 3).

**Fig. 4:**
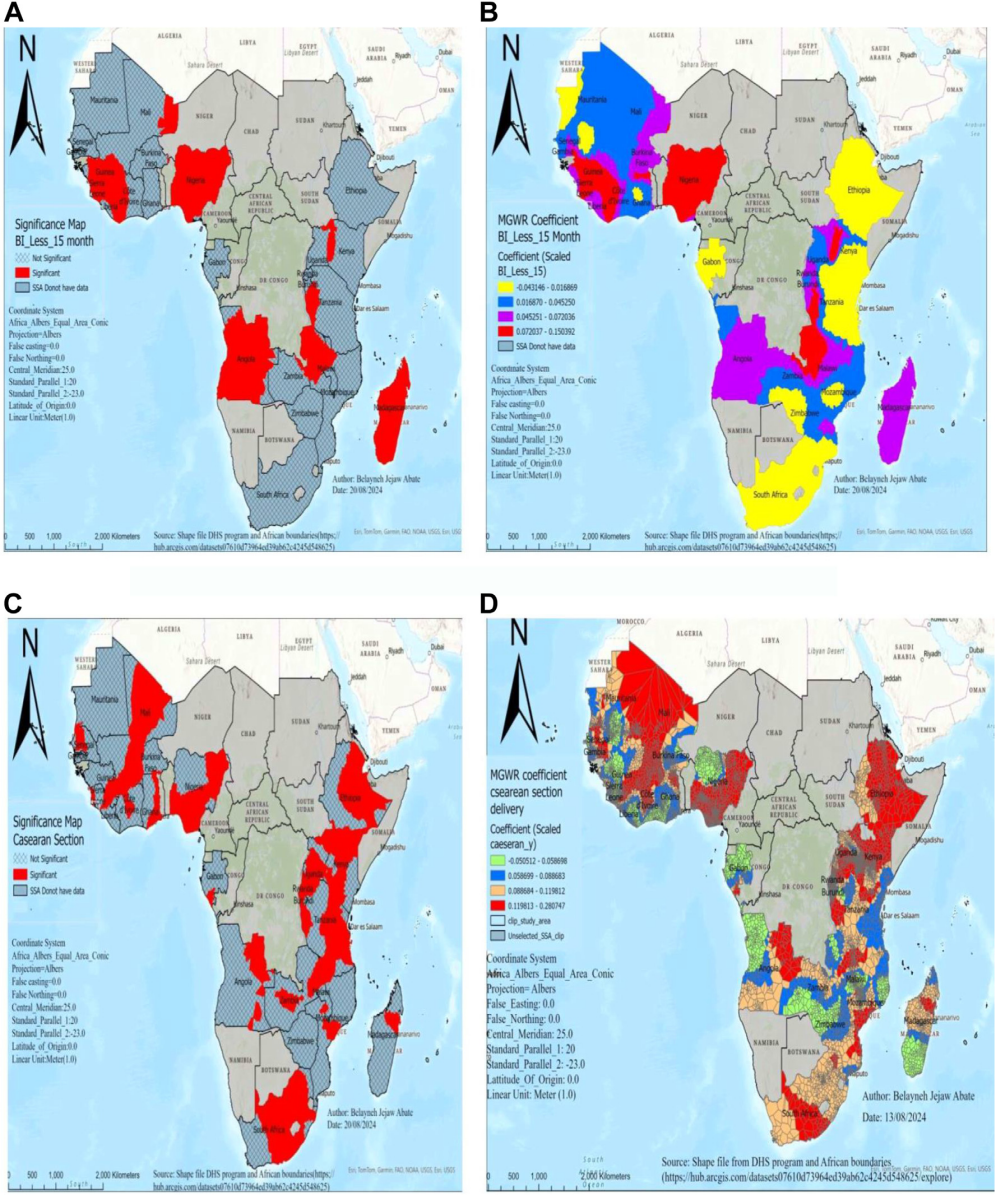
MGWR result of significant areas and coefficients of birth interval less than 15 months (A and B) and cesarean section delivery (C and D) for estimating perinatal mortality in SSA DHS 2015–2023. Notes: Coefficient estimates and significance maps are obtained from MGWR regression. Covariates included are birth interval less than 15 months and women undergoing cesarean section delivery. Red color shades in Fig. B and D indicate countries with high perinatal mortality associated with a birth interval of less than 15 months and women undergoing cesarean section delivery. BI_less_15month denotes a birth interval of less than 15 months, DHS denotes demographic and health survey data, and MGWR represents multi-scale geographic weighted regression.

A 10% increase in birth interval less than 15 months results in a 0.4%–1.9% increase in perinatal mortality in significant areas of Nigeria, Guinea, Sierra Leone, Malawi, and Liberia, while it increased by a range of 0.4%–0.7% in areas of Angola, Madagascar, and some parts of Burundi (Fig. 4A and B).

In significant areas, a 10% increase in women undergoing cesarean section delivery results in a 1.1–2.8% increase in perinatal mortality in areas of Ethiopia, Kenya, Uganda, Mali, Burkina Faso, Nigeria, and South Africa, while it increased by a range of 0.5–0.8% in perinatal mortality in areas of Liberia, Guinea, Sierra Leone, Zambia, and Zimbabwe (Fig. 4C and D).

**Fig. 5:**
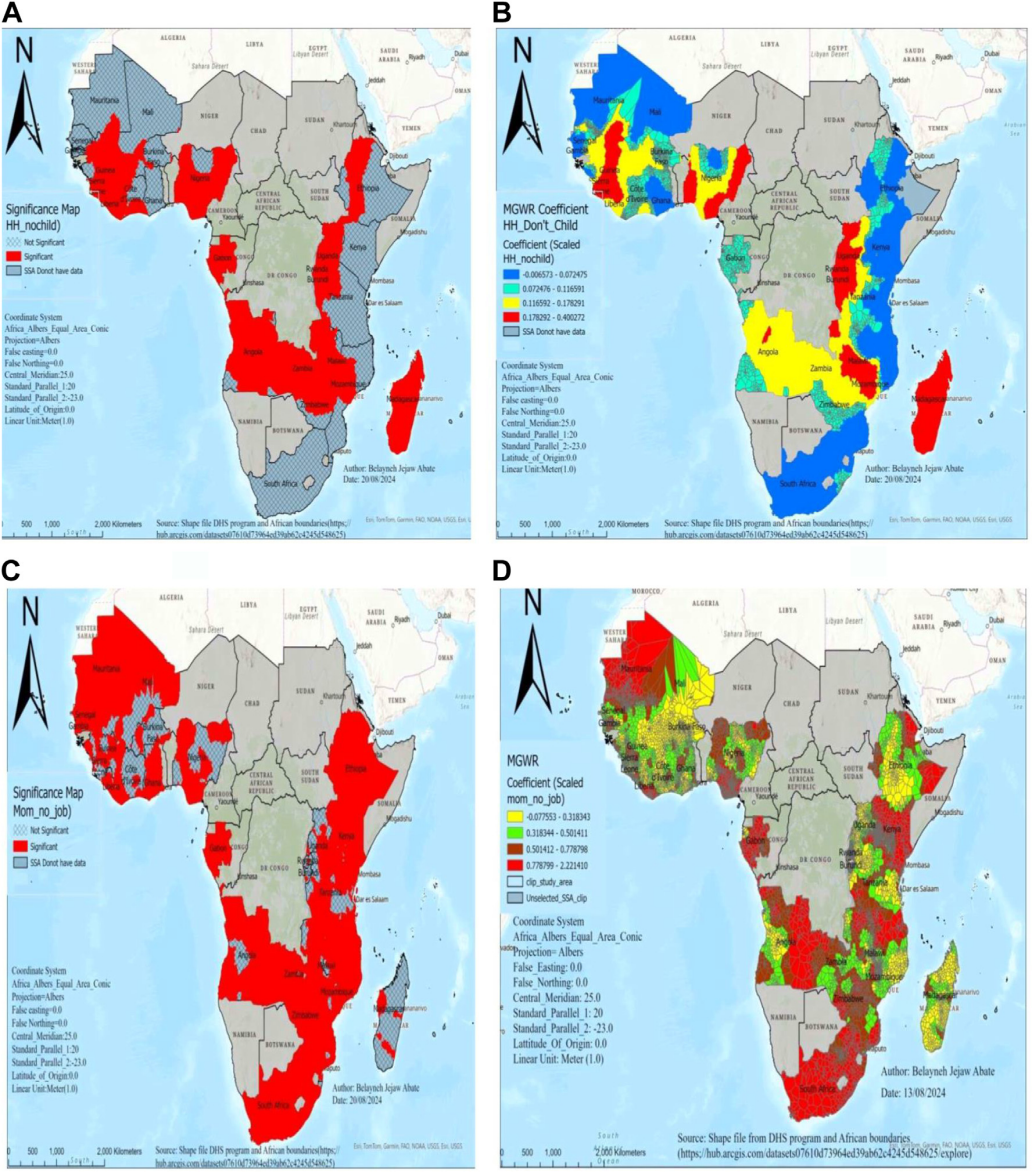
MGWR result of significant areas and coefficients of households without children (A and B) and unemployed women (C and D) for estimating perinatal mortality in SSA DHS 2015–2023. Notes: Coefficient estimates and significance maps are obtained from MGWR regression. Covariates included are households without children and unemployed women. Red color shades in B and D indicate countries with high perinatal mortality associated with households without children and unemployed women. HH_Don’t Child denotes households without children, Mom_No_job denotes unemployed women, DHS denotes demographic and health survey data, and MGWR represents multi-scale geographic weighted regression.

In significant areas, a 10% increase in households without children results in a 1.8–4% rise in perinatal mortality in areas of Uganda, Burundi, Rwanda, Madagascar, Malawi, Mozambique, Guinea, and Sierra Leone, while it increased by a range of 0.7–1.2% in areas of Gabon, Zimbabwe, Côte d’Ivoire, and Ethiopia (Fig. 5A and B).

In statistically significant areas, a 10% increase in unemployed women results in an 8–22.2% rise in perinatal mortality in areas of Kenya, South Africa, Zimbabwe, Gabon, Mauritania, and some parts of Ethiopia, while it increased by a range of 3–5% in areas of Madagascar, Mozambique, and Burkina Faso (Fig. 5C and D).

## Discussion

Spatial analysis is vital for efficiently targeting interventions and designing localized strategies by identifying the most affected areas.^14^ In this study, an attempt has been made to assess the pooled prevalence, spatial variation, and determinants of perinatal mortality in SSA using DHS 2015–2023 data.

Our findings investigated that the pooled prevalence of perinatal mortality in SSA was 46.63 per 1000 total births (95% CI: 42.48, 51.17), which was higher than a previous systematic review that found a rate of 34.7 per 1000 total births.^6^ The discrepancy is likely attributed to variations in the years studied and sample sizes; the current analysis encompasses more recent data from 2015 to 2023 across 25 countries, while the earlier review utilized data from 2010 to 2016 across 21 countries.^6^ Perinatal mortality was highest in West Africa (56 per 1000 total births), followed by Eastern SSA (46/1000), Southern SSA (44/1000), and Central SSA (41/1000). The possible justification might be due to health system challenges, including underfunded healthcare services,^6^ that lead to inadequate prenatal care/postnatal care and poor management of complications, which are crucial for reducing perinatal mortality.^5^^,^^6^ The elevated rate of perinatal mortality implies a critical need for improved health care intervention in SSA.

The spatial analysis identified hotspot areas of perinatal mortality in Nigeria, Madagascar, Rwanda, Malawi, Burundi, Gambia, Uganda, Côte d’Ivoire, Angola, Ethiopia, Burkina Faso, and Senegal, while cold spot areas were found in Kenya, Gabon, South Africa, Ghana, Mali, and Mauritania. This might arise from variations in healthcare access, infrastructure quality, security issues, and education levels that hinder quality of maternal and newborn care.^5^^,^^17^^,^^24^ Additionally, geographical barriers such as seasonal flooding disrupt medical supply chains and limit maternal health service utilization, contributing to poor newborn outcomes in hotspot regions.^25^ Further maternal factors such as malnutrition among pregnant women and complications during pregnancy, including hypertension, diabetes, and infections, are more prevalent in areas with inadequate healthcare services, contributing to higher mortality rates.^5^^,^^7^ Whereas countries with robust health care systems higher maternal education, and successful maternal and child initiatives tends to have low rates of perinatal mortality.^26^^,^^27^ Our findings bring a new evidence and suggest that national health policies should prioritize hotspot regions with higher perinatal mortality rates, allowing for strategic allocation of public health resources to those areas in need.

Our kriging analysis revealed highly predicted areas of perinatal mortality in SSA countries, including Uganda, Kenya, Tanzania, Madagascar, Nigeria, Burkina Faso, Côte d’Ivoire, Sierra Leone, Guinea, Senegal, Angola, Burundi, and Ethiopia. A spatial prediction allows health authorities to pinpoint high-prevalence areas, enabling the allocation of resources and helping to better understand geographic disparities in health outcomes. This insight can inform policymakers about regions requiring immediate attention and tailored strategies. In addition, identifying hotspot areas facilitates community-based initiatives aimed at improving maternal health awareness and access to prenatal care services, guides investments in healthcare infrastructure, and informs policy decisions to address regional disparities that require tailored interventions.

Our study revealed that no ANC visits, birth intervals less than 15 months, women undergoing cesarean section deliveries, households without children, and unemployed women were significant predictors of perinatal mortality.

Countries with higher coefficients of no ANC visits, such as Mali, Burkina Faso, Nigeria, Côte d’Ivoire, Tanzania, Ghana, and Ethiopia, also show hotspots of perinatal mortality. The possible justification might be income-related inequalities affecting access to four or more ANC visits (ANC4+), particularly noted in Burkina Faso, Zambia, Ethiopia, and Zimbabwe,^28^, ^29^, ^30^ and substantial disparities in access to healthcare facilities might hinder pregnant women from obtaining essential ANC services and timely medical interventions.^31^^,^^32^ In addition, religious and social barriers in Tanzania and Malawi, along with geographic and cultural challenges in Malawi, further hinder access to maternal health services, leading to adverse newborn outcomes. This highlights the importance of promoting family planning initiatives.

Regions with higher coefficients of birth intervals less than 15 months, such as Nigeria, Guinea, Sierra Leone, Malawi, and Liberia, also displayed significant hotspots of perinatal mortality. This might be due to the low gross domestic product of the country, poverty, and inadequate family planning. For example, Sierra Leone’s damaged healthcare infrastructure limits contraceptive access and education,^33^ while lower female literacy in Malawi contributes to higher fertility rates and closely spaced pregnancies, leading to higher perinatal mortality rates.^34^^,^^35^ Suggests that health policies should focus on promoting optimal spacing between pregnancies.

Similarly, as the coefficient of women undergoing cesarean section increased, perinatal mortality also increased in regions, including, Ethiopia, Kenya, Uganda, Mali, Burkina Faso, Nigeria, and South Africa. This might be attributed to different factors, like many cesarean section deliveries are performed in emergency situations due to fetal distress or complications, surgical risks associated with C-section, such as infection, and timing of intervention may arise too late in labor, with factors like fetal size, gestational age, and prolonged decision times resulting in adverse newborn outcomes.^36^^,^^37^ In addition, countries like Nigeria, South Africa, and Burkina Faso face extreme heat during the dry season, experiencing dangerous heat waves with high humidity. These conditions can worsen health risks, particularly during cesarean deliveries, by compromising surgical conditions and recovery, leading to adverse outcomes for newborns.^37^ This implies a need for careful evaluation of surgical indications and outcomes.

Areas with the highest coefficient of unemployed women that were positively associated with perinatal mortality were detected in Kenya, South Africa, Zimbabwe, Gabon, Mauritania, and some parts of Ethiopia. This might be variations in economic conditions, educational disparities, social and cultural factors, and geographical barriers across countries that had a significant impact on perinatal mortality.^38^^,^^39^ It indicates that socioeconomic factors play a crucial role in newborns health.

Likewise, households without children were positively associated with increased perinatal mortality rates in areas like Uganda, Burundi, Rwanda, Madagascar, Malawi, Mozambique, Guinea, and Sierra Leone. This might be health crises, economic constraints, and social-cultural factors.^40^^,^^41^ Reveals that targeted interventions may be necessary for specific demographics, including women without children.

This study has several strengths. First, the DHS data is nationally representative and population-based, featuring a large sample size. Second, it illustrates the spatial distribution of perinatal mortality across SSA countries, which can aid in developing localized interventions to achieve the Sustainable Development Goals by 2030. Third, the use of various spatial regression models helps to show the real impact of predictors across each specific geographic area. Whereas this study has certain limitations, first, the DHS data utilized a contraceptive calendar tutor to calculate stillbirths, but twins or triplets are recorded within a single code “B,” which might lead to an undercount of stillbirths,^22^ even though such events are relatively rare. Second, even if we are doing the pooled prevalence, this study doesn’t take into account the time differences of the data collection period across regions of SSA. Third, the geographical locations of enumeration areas were adjusted by up to 2 km in urban areas and 5 km in rural areas to ensure data privacy. This adjustment may influence the estimated effects of spatial analysis within the clusters; to alleviate this problem, buffer analyses were done, so the conclusion or the interpretation should consider the buffer analyses (Appendix 13). Fourth, the cross-sectional design of the study restricts the ability to establish causal relationships between variables and perinatal mortality. Lastly, important explanatory variables such as fetal growth, maternal anemia, obstetric complications like breech delivery, and birth asphyxia were not included in the dataset, limiting the comprehensiveness of the analysis.

In conclusion, perinatal mortality in SSA was high and varied across regions. We identified five predictors that were associated with perinatal mortality that might be a priority for policymakers. Policymakers and other stakeholders, such as maternity and child health programs like WHO, UNICEF, World Bank, and other NGOs, could hold a geographic-based intervention, enhance antenatal care and family planning services, and empower women through employment opportunities to decrease perinatal mortality in the region.

## Contributors

B.J.A.: conceptualization, data duration, formal analysis, methodology, software, and writing–original draft. M.C.A., A.W.M.: writing–review and editing, supervision. H.B.A.: visualization. M.C.A., A.W.M.: accessed and verified the data. B.J.A. and M.C.A. made the decision to submit the manuscript, with B.J.A. serving as the guarantor for the work. All authors read and approved the final version of the manuscript.

## Data sharing statement

The analysis was based on Demographic Health Survey data. Information on the data and content can be available at https://dhsprogram.com/data/available-datasets.cfm.

## Editor note

“The Lancet Group takes a neutral position with respect to territorial claims in published maps and institutional affiliations.”

## Declaration of interests

All authors declare no competing interests.

## Appendix A Supplementary data

Appendices 1–13
