# Supplementary material for: Spatial variation, pooled prevalence, and factors associated with perinatal mortality in Sub-Saharan Africa, evidence from demographic and health surveys 2015–2023: a geospatial regression approach
Source: eClinicalMedicine. 2025 Mar 6;81:103137. doi: 10.1016/j.eclinm.2025.103137 (PMC11929057; doi:10.1016/j.eclinm.2025.103137)
Supplement: Appendices 1–13 [file mmc1.docx]

Appendix 1**:** Sampling procedure and sample size determination for each selected country survey, DHS 2015-2023.

Sub Saharan African Countries (SSA)

(N=46)

SSA countries with DHS datasets

(N= 40)

SSA countries with no DHS datasets

(N=6)

SSA countries with DHS datasets between 2015 and 2023

(N= 27)

SSA countries with DHS datasets earlier than 2015

(N=13)

Total number of selected SSA countries **(n = 25)**

SSA countries which did not have a survey report for perinatal mortality

(N=2)

Enumeration area of selected SSA countries **(n = 15785)**

Selected enumeration area that had latitude and longitude information **(n = 15730)**

Enumeration areas don’t have latitude and longitude information (**n = 55)**

Total number of women aged 15–49 years in selected enumeration area of Sub-Saharan Africa **(n = 392,887)**

Women who had delivered seven or more month’s gestational age, including both stillbirth and live birth, and seven days postpartum (0–6 days) in the 5 years preceding the survey in selected enumeration area of Sub Saharan Africa **(n = 201,566)**

**Figure 1**: Sampling procedure for the study of spatial variation, pooled prevalence, and factors associated with perinatal mortality in Sub-Saharan Africa, DHS 2015-2023.

Notes: Results are obtained from the sampling technique. DHS denotes demographic and health survey, SSA denotes sub-Saharan Africa.

**Sample size determination for each selected country survey**

Weights are vital for ensuring that survey results accurately represent the population, particularly when the sampling design involves disproportionate sampling or varying response rates across different groups. To use weights effectively, first we select the appropriate weight variable (v005) from the DHS IR dataset. Then, we create a new weight variable by dividing v005 by 1,000,000. Finally, when running our analysis, we specify the weight using the `iweight` ([iw = wgt] option, consequently used as a prefix for all analyses done in this study. The final sample sizes for weighted and unweighted samples were presented in the table below **(Table 1)**.

**Table 1: Sample size determination for each selected country survey.**

| Regions | Country | DHS year | Un weighted  Sample size | Weighted  Sample size |
| --- | --- | --- | --- | --- |
| Eastern Africa | Burundi | 2016/17 | 8,945 | 9,253 |
|  | Ethiopia | 2016 | 7,230 | 7,635 |
|  | Kenya | 2022 | 14,654 | 13,849 |
|  | Malawi | 2015/16 | 13,482 | 13,794 |
|  | Mozambique | 2015 | 4047 | 4,234 |
|  | Rwanda | 2021 | 6,396 | 6,543 |
|  | Tanzania | 2022 | 7,950 | 8,132 |
|  | Uganda | 2016 | 10,540 | 10,453 |
|  | Zambia | 2018 | 7,628 | 7,583 |
|  | Zimbabwe | 2015 | 5073 | 5,241 |
| Central Africa | Angola | 2015/16 | 9182 | 8,747 |
|  | Gabon | 2019 | 4,702 | 4,633 |
| Western Africa | Burkina Faso | 2021 | 9,592 | 9,559 |
|  | Ivory Coast | 2021 | 7,985 | 7,536 |
|  | Gambia | 2019 | 6,070 | 5,663 |
|  | Ghana | 2022 | 7,228 | 6,764 |
|  | Guinea | 2018 | 5,785 | 5,753 |
|  | Liberia | 2019/20 | 4,509 | 4,270 |
|  | Mali | 2018 | 6,623 | 6,895 |
|  | Nigeria | 2018 | 22,158 | 22,285 |
|  | Senegal | 2019 | 4,599 | 4,330 |
|  | Sierra Leone | 2019 | 7,763 | 7,636 |
|  | Mauritania | 2021 | 7,842 | 7,928 |
| Southern Africa | South Africa | 2016 | 3,264 | 3,302 |
|  | Madagascar | 2021 | 9,703 | 9,548 |
|  |  |  | 202,950 | 201,566 |

# Appendix 2: Variable measurement.

# Outcome variable measurement

In order to calculate stillbirth, early neonatal mortality, and perinatal mortality, we had used the DHS Contraceptive Calendar tutor [1]; according to the tutor, stillbirths were determined by using information in the calendar, while live births were identified from birth history variables to accurately include twins and triplets, and then stillbirths and live births were summed (assuming all live births meet the gestational age criterion) to determine total births and pregnancies of 7 months or more.

Variables including column 1 of the calendar (vcal_1), year of interview (v007), CMC date of interview (v008), sample weight (v005), row of month of interview (v018), b3 (CMC date of birth), and b6 (age at death) were selected to generate perinatal mortality.

We examine two variables, `beg` and `end`, to designate the start and end positions in the calendar that we want to analyze. The variable `beg` corresponds to the month of the interview, while `end` marks the last month to consider from the five years prior to the interview. Noting that the calendar is in reverse chronological order, where `beg` represents an earlier position and `end` a later one.

Next, we iterate through the entire 80-character calendar in Stata to check for stillbirths during the specified period, using the loop control variable (`i`) as a local macro that applies to all cases and the `in range` function to focus solely on the relevant timeframe. We tally the number of births in the calendar by identifying any character marked as "B"; although this variable is not used since it excludes twins, it serves merely as an example of counting births in the calendar. Similarly, stillbirths were counted by looking for a "T" followed by six "P"s (indicating seven months of pregnancy), and early neonatal deaths and all live births (including twins) are counted by looping through the birth history variables in a similar manner.

We need to set the range of dates to use to limit to the five years preceding the survey, and so we reuse the variables beg and end, this time to specify the beginning and ending century month codes for the period of interest. Sum the total number of pregnancies of 7 or more months and the number of perinatal deaths. In this step we add the births from the birth history (births2) and the stillbirths to give the total number of pregnancies of 7 or more months in the last 5 years in totpreg7m. Similarly, we add the stillbirths and the early neonatal deaths in the last 5 years to calculate all perinatal mortality. Then finally dummy variables were generated for the outcome variable "perinatal mortality" coded as "1" for "yes" and "0" for "no." We define a date range for the five years prior to the survey, reusing the `beg` and `end` variables to specify the relevant century month codes, then calculate the total pregnancies seven or more months by summing births from the birth history and also calculating total perinatal mortality by adding stillbirths and early neonatal deaths, and then finally dummy variables were generated for the outcome variable "perinatal mortality" coded as "1" for "yes" and "0" for "no."

# Explanatory variable measurement

Factors that determine perinatal mortality are organized into four major categories. Their definition and measurement of those variables were adapted from different literature [2-7].

**Table 5: Measurement of explanatory variables used the study.**

| No | Variables | Measurement of variables |
| --- | --- | --- |
| Socio demographic factor | | |
| 201 | Women age in year | Less than 20  20_34  35_44  45_49 |
| 202 | Residence | Urban  Rural |
| 203 | Women education | No education  Primary  Secondary  Post-secondary |
| 204 | Father education | No education  Primary  Secondary  Post-secondary |
| 205 | Sex of household head | Male  Female |
| 206 | Number of children in the house | 0  1_3  4_6 |
| 207 | Mother occupation | Had job  Not had job |
| 208 | Wealth index | Poorest  Poorer  Middle  Richer  Richest |
| Maternal related factor | | |
| 301 | Tetanus toxoid vaccination during pregnancy | Not at all  One times  ≥ 2 + |
| 302 | Antenatal care visits | ≥ 4 visits  1–3 visits  No at all |
| 303 | Women access to decision making | Yes  No |
| 304 | Age at first birth | <20  20-29  30-39  40-49 |
| 305 | Ever had terminated pregnancy | Yes  No |
| Obstetrics related factors | | |
| 401 | Place of delivery | Home  Health facilities |
| 402 | Delivery assistance | Skilled  Non skilled |
| 403 | Mode of delivery | Had caesarean section  No caesarean section |
| 404 | Birth interval |  |
| Environmental related factor | | |
| 501 | Drinking water source | Protected  Unprotected |
| 502 | Toilet facility | Improved  Unimproved |
| 503 | Type of cooking fuel | Clean fuel  Soiled fuel |
| 504 | Distance from health facility  variable | Not big problem  Big problem |
| 505 | Media exposure | Yes  No |

# Appendix 3: Missing data management.

Missing data mechanisms can be classified as one of the following;

- MCAR: Missing completely at random,
- MAR: Missing at random, or
- MNAR: Missing not at random.

Missing data are **MCAR** if the probability of missingness is unrelated to any observed or unobserved data. In other words, the data are MCAR if the reason for missing values in the outcome or predictors has nothing to do with the data values themselves, whether observed or missing. Missing data are **MAR** if the probability of missingness is related to observed data but not to the missing data itself. This means that the missingness can be predicted based on available information. Missing data are **MNAR** if, even given all the observed information, the probability of missingness depends on the unobserved missing values themselves. This means that the missingness cannot be accounted for by any observed data.

Among the 22 predictors, 8 had missing observations. The proportion of missing data was examined by utilizing the ‘mdesc’ package in Stata. The patterns of missing data were assessed descriptively by analyzing the frequency distribution of the predictors, which was somewhat arbitrary **(Table 1).** The types of missing patterns were identified through regression analysis by evaluating the association between the missing variable and other explanatory variables, and it was missing at random and managed according to DHS guidelines **(Table 2).**

**Table 1: Missing-value patterns (1 means complete).**

| Percent | 1 | 2 | 3 | 4 | 5 | 6 | 7 | 8 |
| --- | --- | --- | --- | --- | --- | --- | --- | --- |
| 62% | 1 | 1 | 1 | 1 | 1 | 1 | 1 | 1 |
| 13 | 1 | 1 | 1 | 1 | 1 | 1 | 1 | 0 |
| 7 | 1 | 1 | 1 | 1 | 1 | 1 | 0 | 1 |
| 6 | 1 | 1 | 1 | 1 | 1 | 1 | 0 | 0 |
| 5 | 1 | 1 | 0 | 0 | 0 | 0 | 1 | 1 |
| 2 | 1 | 0 | 1 | 1 | 1 | 1 | 1 | 1 |
| 1 | 1 | 1 | 0 | 0 | 0 | 0 | 1 | 0 |
| <1 | 1 | 1 | 0 | 0 | 0 | 0 | 0 | 1 |
| <1 | 1 | 0 | 1 | 1 | 1 | 1 | 1 | 0 |
| <1 | 0 | 1 | 1 | 1 | 1 | 1 | 1 | 1 |
| <1 | 1 | 1 | 0 | 0 | 0 | 0 | 0 | 0 |
| <1 | 0 | 1 | 1 | 1 | 1 | 1 | 0 | 1 |
| <1 | 0 | 1 | 1 | 1 | 1 | 1 | 0 | 0 |
| <1 | 1 | 1 | 1 | 1 | 0 | 1 | 1 | 1 |
| <1 | 0 | 1 | 1 | 1 | 1 | 1 | 1 | 0 |
| <1 | 1 | 1 | 1 | 1 | 1 | 0 | 1 | 1 |
| <1 | 1 | 0 | 1 | 1 | 1 | 1 | 0 | 0 |
| <1 | 1 | 0 | 1 | 1 | 1 | 1 | 0 | 1 |
| <1 | 1 | 1 | 1 | 1 | 1 | 0 | 1 | 0 |
| <1 | 1 | 1 | 1 | 1 | 0 | 1 | 1 | 0 |
| <1 | 1 | 1 | 1 | 1 | 1 | 0 | 0 | 0 |
| <1 | 1 | 0 | 0 | 0 | 0 | 0 | 1 | 0 |
| <1 | 1 | 1 | 1 | 1 | 1 | 0 | 0 | 1 |
| <1 | 1 | 1 | 1 | 1 | 0 | 1 | 0 | 0 |
| <1 | 1 | 1 | 1 | 1 | 0 | 1 | 0 | 1 |
| <1 | 1 | 0 | 0 | 0 | 0 | 0 | 1 | 1 |
| <1 | 1 | 0 | 0 | 0 | 0 | 0 | 0 | 0 |
| <1 | 1 | 0 | 1 | 1 | 0 | 1 | 1 | 1 |
| <1 | 0 | 1 | 0 | 0 | 0 | 0 | 1 | 1 |
| <1 | 0 | 1 | 0 | 0 | 0 | 0 | 0 | 0 |
| <1 | 0 | 1 | 0 | 0 | 0 | 0 | 1 | 0 |
| <1 | 1 | 0 | 0 | 0 | 0 | 0 | 0 | 1 |
| <1 | 1 | 0 | 1 | 1 | 0 | 1 | 1 | 0 |
| <1 | 0 | 0 | 1 | 1 | 1 | 1 | 1 | 0 |
| <1 | 0 | 1 | 0 | 0 | 0 | 0 | 0 | 1 |
| <1 | 0 | 1 | 1 | 1 | 0 | 1 | 1 | 1 |
| <1 | 1 | 1 | 1 | 1 | 0 | 0 | 1 | 1 |
| 100% |  |  |  |  |  |  |  |  |

Variables are distance from health facility (1), women occupation (2), ANC visit (3), place of delivery (4), cesarean section delivery (5), TT vaccine (6), husband education (7), and preceding birth interval (8).

## Table 2: Missing data management.

| Variables | Missing | Total | Percent missing | Missing data management according to DHS guideline |
| --- | --- | --- | --- | --- |
| Sex of household head | 0 | 202,950 | 0.00 | Don’t have missing value |
| Women age | 0 | 202,950 | 0.00 | Don’t have missing value |
| Place of residence | 0 | 202,950 | 0.00 | Don’t have missing value |
| Women education level | 0 | 202,950 | 0.00 | Don’t have missing value |
| Number of children in the house | 0 | 202,950 | 0.00 | Don’t have missing value |
| Mother occupation | 6900 | 202,950 | 3.4 | Included as no occupation |
| Father education | 8320 | 202,950 | 4.1 | Included as no education |
| Wealth index | 0 | 202,950 | 0.00 | Don’t have missing value |
| Place of delivery | 5886 | 202,950 | 2.96 | A separate category for missing values, but very low percentage then categorized into home |
| Assistance during delivery | 0 | 202,950 | 0.00 | Don’t have missing value |
| Delivery by cesarean section | 6311 | 202,950 | 3.11 | Assumed not Caesarean section |
| ANC visit during pregnancy | 9132 | 202,950 | 4.5 | Grouped into categories of no antenatal visit |
| TT vaccine | 6698 | 202,950 | 3.3 | It was treated as 0 injections |
| Ever had terminated pregnancy | 0 | 202,950 | 0.00 | Don’t have missing value |
| Women access to decision making | 0 | 202,950 | 0.00 | Don’t have missing value |
| Birth interval | 12989 | 202,950 | 6.4 | Birth dates of children are imputed if missing |
| Media Exposure | 3 | 202,950 | 0.00 | Included in no media exposure |
| Source of drinking water | 0 | 202,950 | 0.00 | Don’t have missing value |
| Type of toilet facility | 0 | 202,950 | 0.00 | Don’t have missing value |
| Type of cooking fuel | 0 | 202,950 | 0.00 | Don’t have missing value |
| Distance from health facility | 5479 | 202,950 | 2.7 | It was categorized as no big problem |

# Appendix 4: Map projection.

# Figure 1: Map projection selection for the studies of spatial variation and its associated factor of perinatal mortality in SSA: DHS 2015-2023.


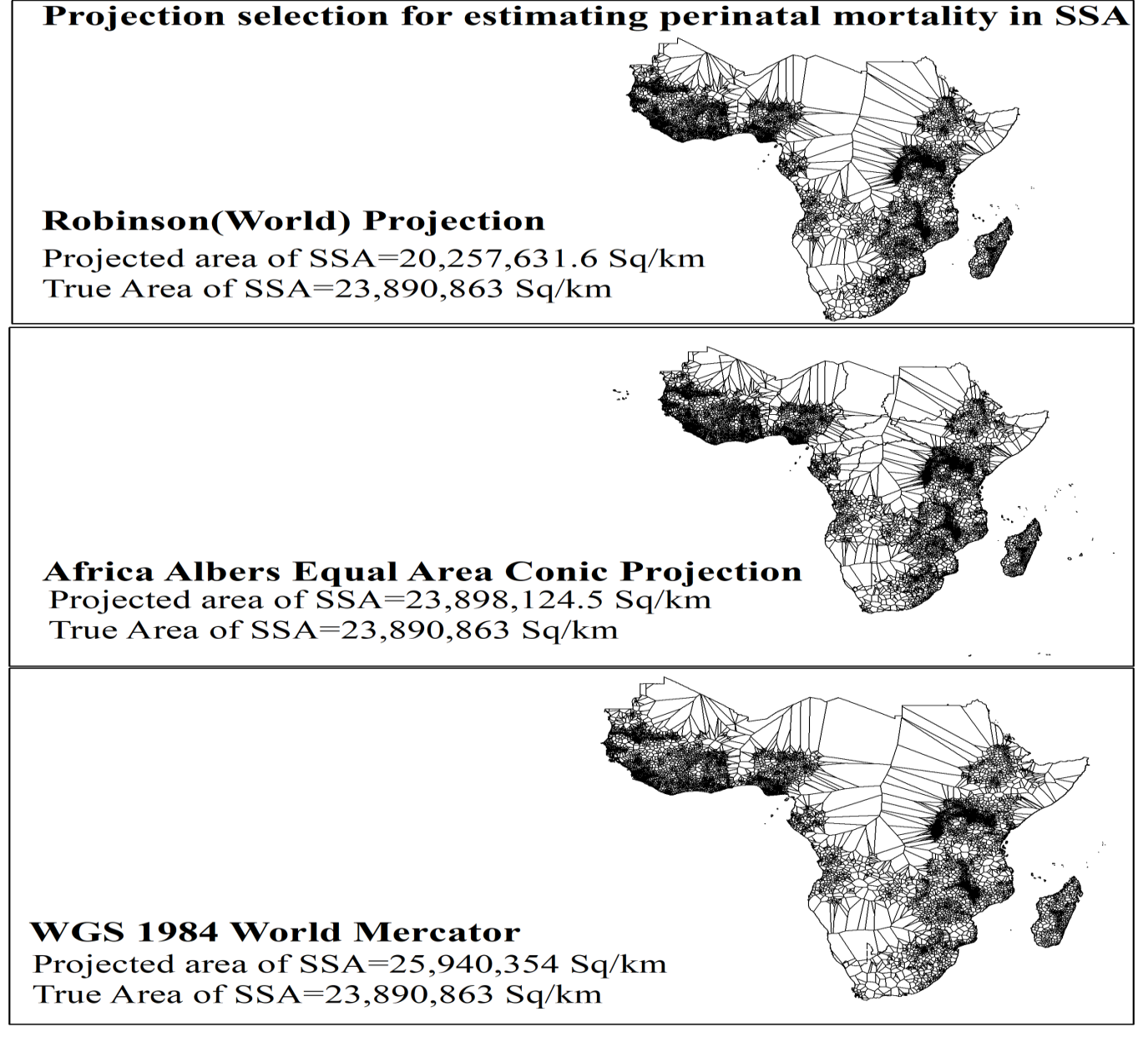


Notes: Results are obtained from map projection selection. DHS denotes demographic and health survey, SSA denotes sub-Saharan Africa, WGS denotes world geodetic system, and sq/km denotes square/kilometer.

**Appendix 5: Variable transformation.**

Figure 1: A-D different types of transformation to ensure the normality assumption for the study of spatial distribution and associated factors of perinatal mortality in SSA: DHS 2015-2023.


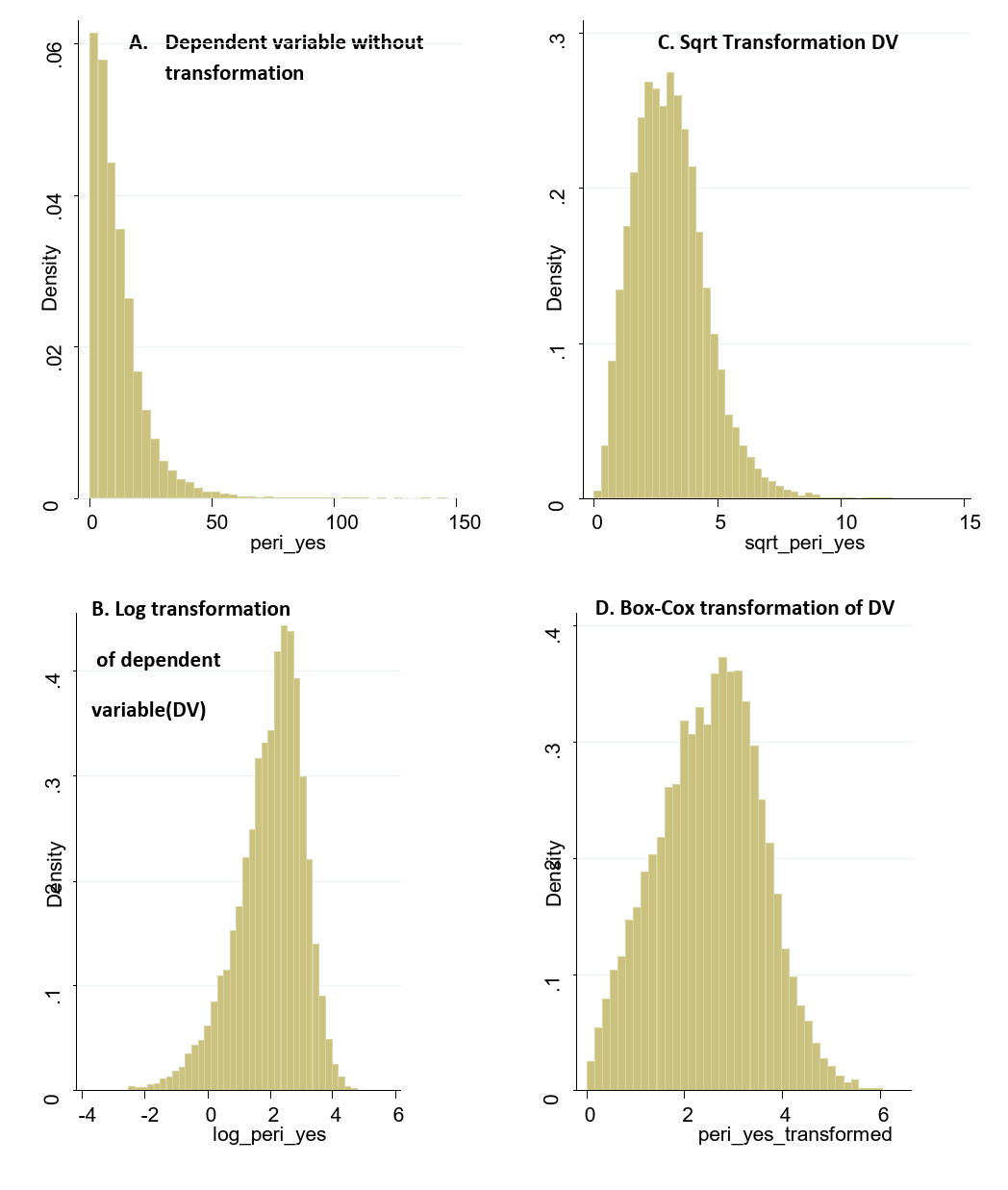
 Notes: Results are obtained from outcome variable transformation. DV denotes dependent variable, sqrt_peri_yes stands for square root transformation of perinatal mortality, log_peri_yes denotes log transformation of perinatal mortality, peri_yes_transformed denotes box cox transformation result of perinatal mortality, and peri_yes represents perinatal mortality without any transformation.

# Appendix 6: Exploratory regression.

Exploratory regression is a valuable tool in fulfilling the assumptions of Ordinary Least Squares (OLS) regression by iterating through all possible combinations of candidate explanatory variables to identify the best-fitting model that meets OLS criteria. It also increases the likelihood of finding a model that not only fits well but also adheres to the necessary statistical conditions for reliable inference.

Exploratory regression revealed the statistical significance of each explanatory variable, with a summary provided in **Table 1;** 15 variables with over 75% significance were selected as a good candidate for OLS. After examining different combinations, we identified 5 predictors (no ANC visit, birth interval less than 15 months, women undergoing cesarean section delivery, unemployed women, and households without children). The predictors were selected because they had statistically significant coefficients (P value < 0.01), an adjusted R²=54, a multicolinearity condition number of 2.72, a Jarque bera test of 0.95, and a Koenker-Basset of 0.000 and also those predictors acted as a diagnostic tool for other additional models.

**Table 1:** Exploratory regression for identification of good candidate explanatory variables for the study of spatial distribution and its associated factor of perinatal mortality in SSA: DHS 2015-2023.

| Variable | % Significant | % Negative | % Positive |
| --- | --- | --- | --- |
| No ANC at all | 100 | 0.00 | 100 |
| Birth interval less than 15 month | 100 | 0.00 | 100 |
| Mom no job | 100 | 0.00 | 100 |
| Women undergoing CS | 100 | 0.00 | 100 |
| Terminated pregnancy | 100 | 0.00 | 100 |
| No child in the Household | 100 | 0.00 | 100 |
| Women no education | 100 | 0.00 | 100 |
| Household head male | 100 | 0.00 | 100 |
| Timing ANC above 12 week | 100 | 0.00 | 100 |
| Home delivery | 95.81 | 49.71 | 50.29 |
| No media Exposure | 86.55 | 8.77 | 91.23 |
| Unskilled Delivery Assistant | 85.48 | 46.98 | 53.02 |
| Poorer | 79.04 | 35.19 | 64.81 |
| TT vaccine not at all | 77.10 | 56.73 | 43.27 |
| Rural Residence | 75.34 | 12.96 | 87.04 |

Notes: Results are obtained from exploratory regression. ANC represents antenatal care, CS represents cesarean section, and TT represents tetanus.

# Appendix 7: Decision tree for global regression model.

Figure 1: Decision tree of global regression model for determining perinatal mortality in SSA: DHS 2015-2023 [8].


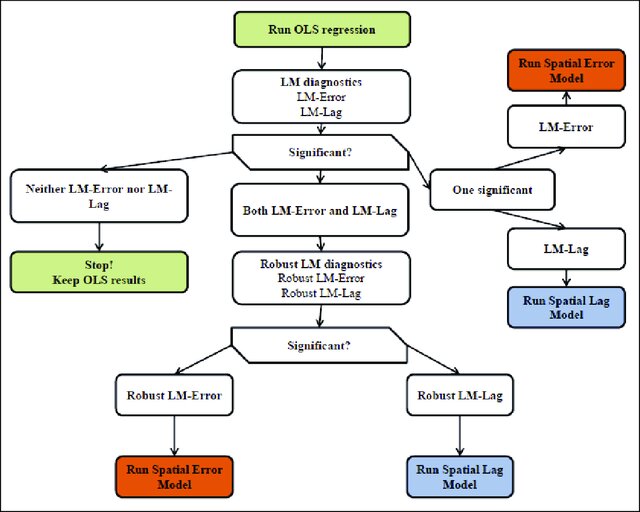


Notes: Results are obtained from global regression model. OLS denotes ordinary least square, LM denotes Lag range multiplier.

# Appendix 8

1. **Level of Analysis**

We have indicated that the unit of analysis is at the level of enumeration areas within the 25 Sub-Saharan African countries included in our study.

To clarify, before conducting the spatial analysis, we obtained shape files containing geographic coordinates (latitude and longitude) for the enumeration areas from the MEASURE DHS program. Additionally, we downloaded boundary files for Africa from the African shapefile boundaries repository. We then aggregated the enumeration areas based on their cluster numbers (specifically V001), using the latitude and longitude data.

For example, as illustrated in the Table 1 below, we processed the first 20 enumeration areas (EA) for the outcome variable using STATA software. In this analysis, perinatal mortality was coded as 0 for "no" and 1 for "yes." We identified perinatal mortality rates using weighted frequencies for each cluster, applying the same procedure across all EAs.

**Table 1**: Sample data extraction for geospatial analysis of perinatal mortality in 20 enumeration areas of SSA countries, DHS 2015-2023.

| v001 | 0 | 1 | Total |
| --- | --- | --- | --- |
| 1 | 11.277256 | 0 | 11.277256 |
| 2 | 5.781366 | 0 | 5.781366 |
| 3 | 11.904809 | 0 | 11.904809 |
| 4 | 10.442674 | 0 | 10.442674 |
| 5 | 11.598688 | 0 | 11.598688 |
| 6 | 23.034396 | 0 | 23.034396 |
| 7 | 16.769056 | 0 | 16.769056 |
| 8 | 19.62734 | 1.962734 | 21.590074 |
| 9 | 18.4755 | 0 | 18.4755 |
| 10 | 15.41272 | 0 | 15.41272 |
| 11 | 23.682233 | 0 | 23.682233 |
| 12 | 16.196067 | 0 | 16.196067 |
| 13 | 9.3250022 | 0 | 9.3250022 |
| 14 | 10.527272 | 0 | 10.527272 |
| 15 | 21.969659 | 8.7878637 | 30.757523 |
| 16 | .218228 | 0 | .218228 |
| 17 | 27.54946 | 5.509892 | 33.059352 |
| 18 | 26.429479 | 0 | 26.429479 |
| 19 | 17.524223 | 0 | 17.524223 |
| 20 | 12.569848 | 0 | 12.569848 |
| Total | 310.31528 | 16.2604897 | 326.57577 |

Notes: Results are obtained from sample data extraction in STATA software for only 20 enumeration areas. V001 denotes the cluster number for each country. 0 represents no perinatal mortality. 1 represents perinatal mortality.

Each enumeration area corresponds to specific countries; for instance, the Ethiopia DHS 2016 dataset contains 645 enumeration areas. If perinatal mortalities were recorded in these enumeration areas, we identified them as hotspots, cold spots, or significant predictors based on the context of our geospatial analysis. In total, we utilized 15,785 enumeration areas from the 25 Sub-Saharan African countries.

To avoid confusion since each country's EAs start at number one, we renamed the enumeration areas sequentially. For example, if Ethiopia was the first country aggregated, we numbered its enumeration areas from 1 to 645, and the next country would start at 646. This method was consistently applied to all selected countries.

Using this systematic approach, we identified hotspots, cold spots, outliers, most likely cluster, and significant predictors of perinatal mortality within these geographic areas based on specific geospatial analysis that we used. These findings were visually represented using ArcGIS software by incorporating African boundary shape files. When significant outcomes emerged in specific countries or regions on the map, we interpreted these as indicative of particular events occurring in those areas.

Our interpretation was grounded in both the enumerated geographic units (EAs) and the visualized patterns of perinatal mortality on the map. This integrated approach allowed us to provide nuanced insights into how perinatal mortality varies across different regions within Sub-Saharan Africa.

# Appendix 8

# Working flow and description of spatial regression models

Figure 1: Illustrating the workflow of the study for the study of perinatal mortality in SSA: DHS 2015-2023.

- Socio demographic characteristics
- Maternal and child health related factor
- Obstetrics related factor
- Environmental related factor

**Independent variable**

**Outcome variable**

- Perinatal mortality

**Select variables**

- Exploratory regression

OLS

- Regression Diagnostic (variance inflation factor (VIF), Jarque bera test)
- Diagnostic for heteroskedasticity ( koenker test)
- Diagnostic for spatial dependence (LM test)

Adj R2

AICc

Best fit model coefficients

Notes: Results are obtained from global and local regression models. OLS = Ordinary Least Square, LM = Lag range Multiplier, SLM = Spatial Lag Model, SEM = Spatial Error Model

# Description of spatial regression models

### **Ordinary least squares (OLS) regression**.

The ordinary least squares (OLS) regression method is used to investigate the relationships between a set of explanatory variables and perinatal mortality. It can be expressed in a form [8].

$$yi=\beta0+xi\beta+£i$$

Where yi is the perinatal mortality rate for county i, β0 is the intercept, xi is the vector of explanatory variables, β is the vector of regression coefficients, and εi is the random error term.

Even if the exploratory regression identifies the best candidate five covariates for OLS, the assumption of spatial independence may be violated in scenarios of spatial autocorrelation (SA) that would result in underestimation of standard errors and overstated significance testing related to inappropriate inference processes. To address this issue, this study employed the SLM and SEM models.

### **Spatial lag model**

The SLM assumes dependency between the dependent variables in neighbors and incorporates spatial dependency between the parameters into the regression model that helps us to examine how the perinatal mortality rate is influenced by adjacent neighborhoods. In this study, a spatial lag model assumes that there could be perinatal mortality dependencies among the clusters of SSA. A positive value of spatially lagged perinatal mortality implies that countries are expected to have higher rates of perinatal mortality if, on average, their neighbors have higher perinatal mortality.

SLM is denoted by:

$$yi= \rho Wy+\beta X+ \varepsilon j$$

Where $y$ is the dependent variable for county i, ρ is the coefficient associated with the spatially lagged dependent variable, Wy denotes the spatially lagged dependent variable (spatially lagged perinatal mortality), β denotes the coefficient associated with X, X is the predictor variable, and εj is the error term [8].

### **Spatial error model**

The spatial error model (SEM) assumes that errors are correlated with the errors in neighboring locations. In other words, the SEM captures the spatial autocorrelation in the residuals, which is not addressed by the standard OLS regression. In addition, it takes into account unobserved spatial factors that influence the error terms in regression analysis, recognizing the impact of omitted variables on spatial dependence [8].

A spatial error model (SEM) can be represented in the following manner:

$$yi=\beta Xj+ \rho\sum_{j\neq1} WijYj+ \varepsilon j$$

Where $yi$ denotes the prevalence of perinatal mortality for the i-th country, Wij indicates the spatial weight of proximity between country i and j, Yj is the prevalence of perinatal mortality in the j-th country, βj denotes the coefficient, Xj is the predictor variable, and i is the residual. However, those global models don’t show the spatial relationship of perinatal mortality across space.

**Geographically Weighted Regression (GWR)**

These methods helped us capture spatial non-stationarity by allowing regression coefficients to vary across different uniform spatial scaling units. A positive coefficient implies that the predictor and perinatal mortality are in the same direction, while a negative coefficient stands for the opposite (63).

The GWR model can be written as:

$$yi=\beta i0+\sum_{j=1}^{m} \beta ijXij+\varepsilon i. i=1 2 3\ldots n$$

Where at county i, yi is the value for the perinatal mortality rate, βi0 is the intercept, βij is the jth regression parameter, Xij is the value of the jth explanatory parameter, and εi is a random error term [9].

**Multi-Scale Geographically Weighted Regression (MGWR)**

By employing MGWR, we addressed the limitation of uniform spatial scaling, thus providing a more accurate representation of relationships between predictors and perinatal mortality. The bandwidth for each of the predictors is determined individually using the golden section search rather than assuming a uniform spatial scale for all the covariates. This approach allows the model to capture various scales of relationship non-stationarity for every target-to-predictor variable linkage [10], while MGWR also adopts an iterative back-fitting procedure when model calibration is considered, which extends the GWR framework to further account for different processes that may operate at different spatial scales within the study area.

MGWR can be formulated as

$$yi=\sum_{j=1}^{m} \beta bwjXij+\varepsilon i. i=1 2 3\ldots n$$

Where βbwj is the bandwidth used for calibration of the jth relationship, and the rest of the parameters are the same as the standard GWR approach.

# Appendix 9: Spatial autocorrelation (A) and high-low clustering report (B)


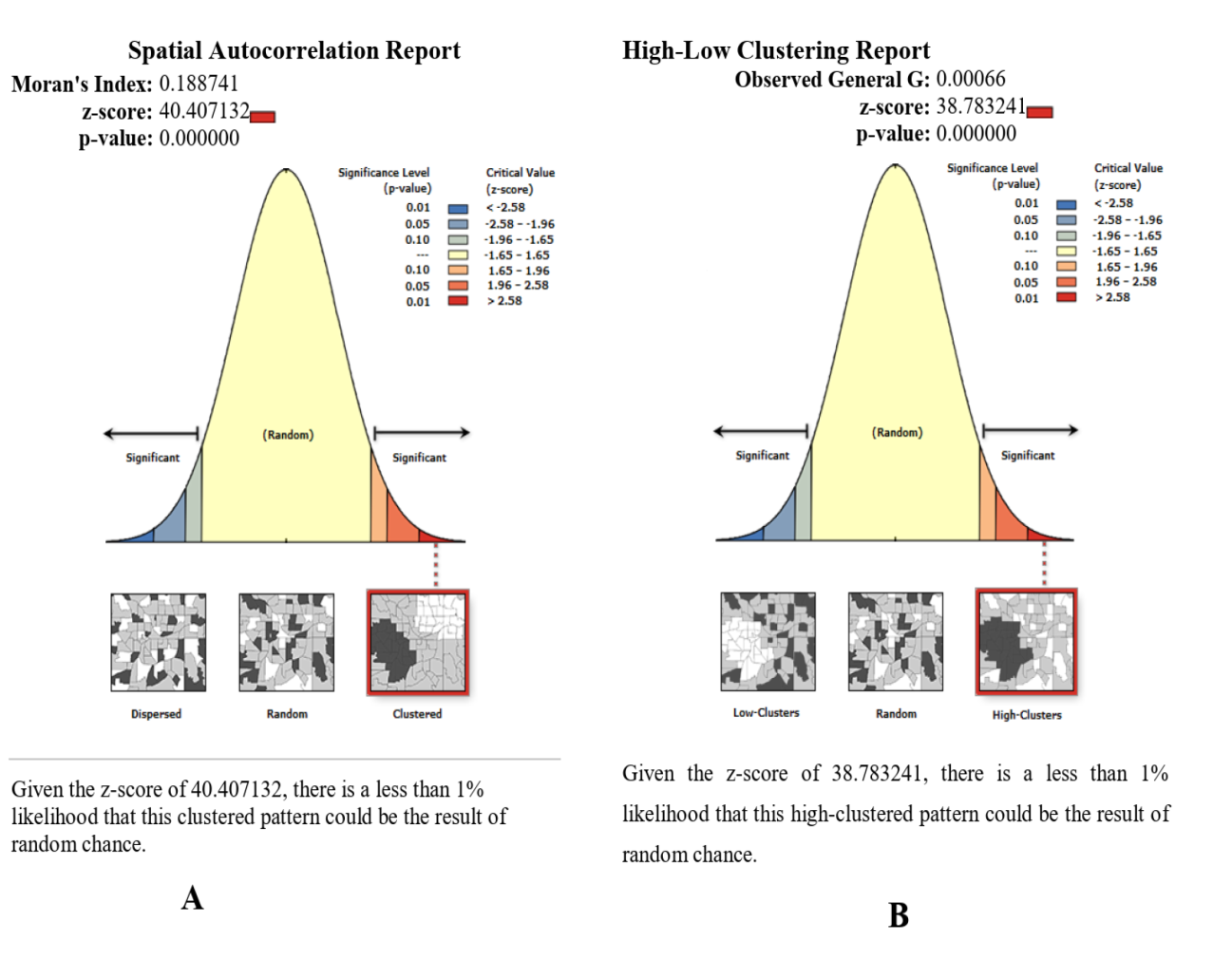
Appendix 10: SatScan Analysis

Table 1: Significant SatScan analysis of perinatal mortality in SSA: DHS 2015-2023.

| Cluster type | Number of location identified | Coordinates/radius km | Population | Cases | RR | LLR | P-value |
| --- | --- | --- | --- | --- | --- | --- | --- |
| Primary(most likely) | 678 | 11.19038N, 7.8251136E/475.39km | 13259 | 1088 | 1.83 | 158.6 | P<0.01 |
| Secondary(1^st^ cluster) | 137 | 1.415116N, 30.87648E/ 179.7km | 2261 | 179 | 1.68 | 21.4 | P<0.01 |
| Secondary(2^nd^ cluster) | 65 | -13.02157N, 14.98804E/ 69.4km | 1505 | 131 | 1.85 | 21.3 | P<0.01 |
| Secondary(3^rd^ cluster) | 2 | 7.887302N, 39.86272E/ 29.71km | 77 | 20 | 5.48 | 19.6 | P<0.01 |
| Secondary(4^th^ cluster) | 1 | -13.91513N, 33.35088E/0km | 60 | 16 | 5.63 | 16.1 | P<0.01 |
| Secondary(5^th^ cluster) | 38 | -20.02374N, 33.01289E/142.9km | 464 | 49 | 2.23 | 13.1 | 0.02 |
| Secondary(6^th^ cluster) | 3 | -6.221925N, 35.75617E/5.74km | 65 | 15 | 4.86 | 21.4 | 0.02 |

Notes: Results are obtained from SatScan analysis. RR = relative risk, LLR = log likelihood ratio

# Appendix 11: Model diagnosis for global regression model

Table 1: Diagnostic test for global regression models to determine factors associated with perinatal mortality in SSA: DHS 2015-2023.

| Regression Diagnostics | | | |
| --- | --- | --- | --- |
| Multicollinarity condition number | 2.724440 | | |
| Test on normality of error | | | |
| Test | DF | Value | Probability |
| Jarque bera test | 2 | 0.1008 | 0.95084 |
| Diagnostic for heteroskedascity | | | |
| Test | DF | Value | Probability |
| Breush-Pagan test | 5 | 752.9268 | P <0.01 |
| Koenker Bassett test | 5 | 752.2447 | P <0.01 |
| Diagnostics for spatial dependence | | | |
| Test | MI/DF | Value | Probability |
| Moran’s I (error) | 0.5606 | 112.4067 | P <0.01 |
| Lagrange Multiplier (Lag) | 1 | 9386.0432 | P <0.01 |
| Robust LM (Lag) | 1 | 590.3415 | P <0.01 |
| Lagrange Multiplier (Error) | 1 | 12617.4248 | P <0.01 |
| Robust LM (Error) | 1 | 3821.7230 | P <0.01 |
| Lagrange Multiplier (SARMA) | 1 | 13207.7662 | P <0.01 |

Notes: Results are obtained from regression diagnostics. MI = Moran’s I, DF = Degree of freedom

# Appendix 12: Performance comparison for global and local model regression

Table 1: Performance of global and local regression models for estimating perinatal mortality in SSA, DHS 2015-2023.

| Category | OLS | SLM | SEM | GWR | MGWR |
| --- | --- | --- | --- | --- | --- |
| $\mathbf{Adjusted R}^{\mathbf{2}}$ | 54.7% | 74% | 78.6% | 80.5 | 85.6 |
| AICc | 33887.8 | 26274.3 | 24466 | 21712.08 | 16460.4 |

Notes: Results are obtained from global and local regression models. OLS = Ordinary Least Square, SLM = Spatial Lag Model, SEM = Spatial Error Model, GWR = Geographic Weighted Regression, MGWR = Multi-scale Geographic Weighted Regression.

# Appendix 13: Buffer analysis

# Figure 1: Buffer analysis for the studies of spatial variation and its associated factor of perinatal mortality in SSA: DHS 2015-2023.


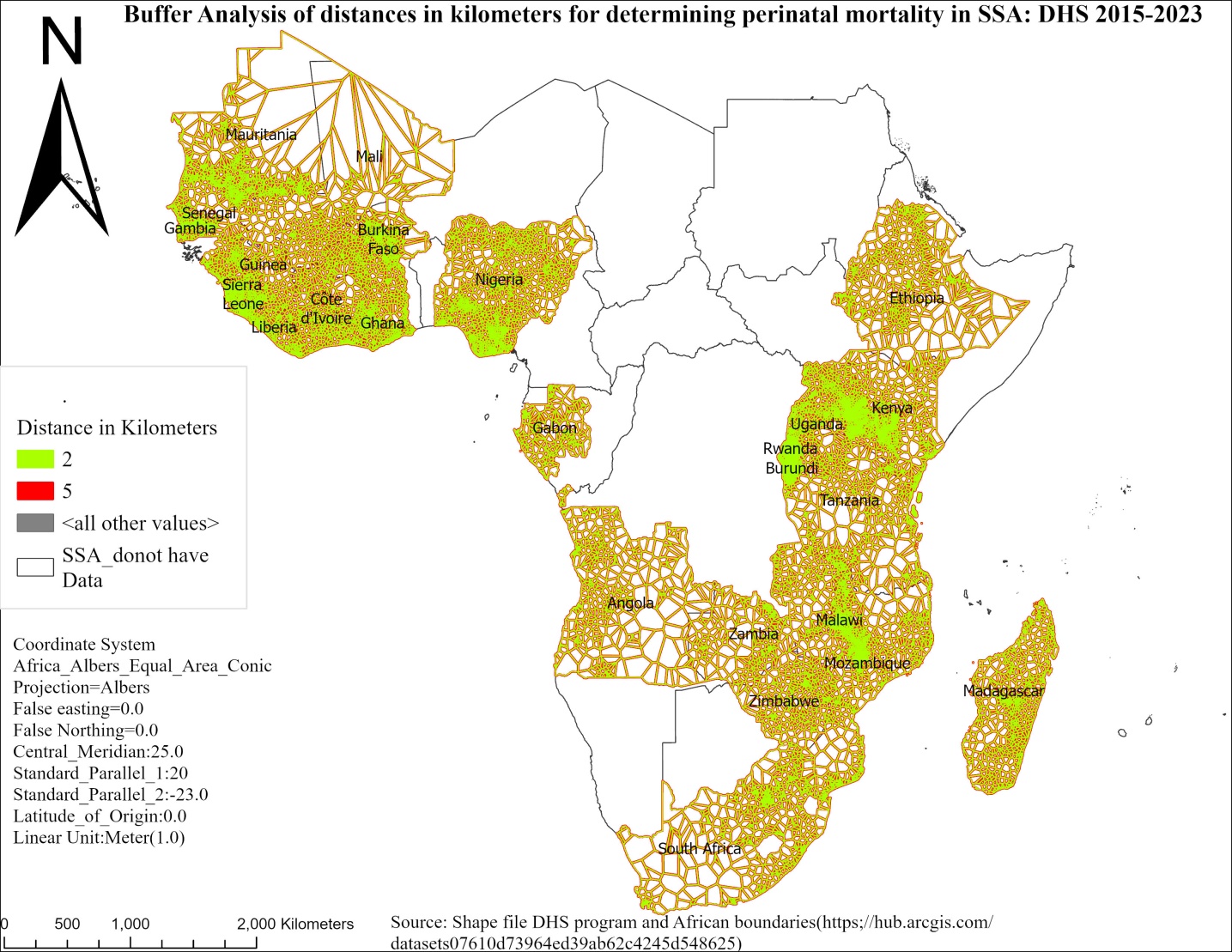


Resultgs are obtained from buffer analys. Red color shades indicates countries that had collect geographic coordinate data within 5 kilo meter. DHS; denotes demographic and health survey data, SSA; denotes Sub Saharan Africa

**References**

1. CALENDARS, I., *DHS METHODOLOGICAL REPORTS 25.* 2018.

2. Ezeh, O.K., et al., *Community-and proximate-level factors associated with perinatal mortality in Nigeria: evidence from a nationwide household survey.* BMC Public Health, 2019. **19**: p. 1-9.

3. Hossain, M.B., et al., *Trends and determinants of perinatal mortality in Bangladesh.* PloS one, 2019. **14**(8): p. e0221503.

4. Girma, D., et al., *Individual and community-level factors of perinatal mortality in the high mortality regions of Ethiopia: a multilevel mixed-effect analysis.* BMC Public Health, 2022. **22**(1): p. 247.

5. chanie, m., et al., *Trend, multivariate decomposition and spatial distribution of perinatal mortality in Ethiopia using further analysis of EDHS 2005-2016.* medRxiv, 2023: p. 2023.07. 25.23293164.

6. Ghimire, P.R., et al., *Factors associated with perinatal mortality in Nepal: evidence from Nepal demographic and health survey 2001–2016.* BMC pregnancy and childbirth, 2019. **19**: p. 1-12.

7. Yadeta, T.A., et al., *Spatial pattern of perinatal mortality and its determinants in Ethiopia: Data from Ethiopian Demographic and Health Survey 2016.* PLoS One, 2020. **15**(11): p. e0242499.

8. Chaurasia, H., et al., *Does seasonal variation affect diarrhoea prevalence among children in India? An analysis based on spatial regression models.* 2020. **118**: p. 105453.

9. Mollalo, A., B. Vahedi, and K.M.J.S.o.t.t.e. Rivera, *GIS-based spatial modeling of COVID-19 incidence rate in the continental United States.* 2020. **728**: p. 138884.

10. Seboka, B.T., et al., *Spatial trends and projections of chronic malnutrition among children under 5 years of age in Ethiopia from 2011 to 2019: a geographically weighted regression analysis.* 2022. **41**(1): p. 28.
